# Prognostic Value of Bone Mineral Density on Curve Progression: A Longitudinal Cohort Study of 513 Girls with Adolescent Idiopathic Scoliosis

**DOI:** 10.1038/srep39220

**Published:** 2016-12-19

**Authors:** Benjamin Hon Kei YIP, Fiona Wai Ping YU, Zhiwei WANG, Vivian Wing Yin HUNG, Tsz Ping LAM, Bobby Kin Wah NG, Feng ZHU, Jack Chun Yiu CHENG

**Affiliations:** 1 Department of Orthopaedics and Traumatology, Faculty of Medicine, Prince of Wales Hospital, The Chinese University of Hong Kong, Hong Kong; 2 JC School of Public Health and Primary Care, Faculty of Medicine, The Chinese University of Hong Kong, Hong Kong; 3 Joint Scoliosis Research Center of the Chinese University of Hong Kong and Nanjing University, Hong Kong; 4 Bone Quality and Health Centre, Department of Orthopaedics and Traumatology, Faculty of Medicine, Prince of Wales Hospital, The Chinese University of Hong Kong, Hong Kong; 5 SH Ho Scoliosis Research Laboratory, Faculty of Medicine, Prince of Wales Hospital, The Chinese University of Hong Kong, Hong Kong; 6 Department of Orthopedic Surgery, Second Affiliated Hospital, School of Medicine, Zhejiang University, Hangzhou, China; 7 Spine Surgery, the Affiliated Drum Tower Hospital of Nanjing University Medical School, Nanjing, China

## Abstract

Osteopenia has been found to occur in about 30% of Adolescent Idiopathic Scoliosis (AIS) patients. This study aimed to investigate its prognostic value on the risk of curve progression to surgical threshold. Newly diagnosed AIS girls (N = 513) with Cobb angle 10°–40° were recruited with follow-up till maturity. Bilateral hips were assessed with dual-energy x-ray absorptiometry (DXA). Distal radius of a subgroup of 90 subjects was further assessed with high-resolution peripheral quantitative computed tomography (HR-pQCT). 55 patients progressed to surgical threshold or underwent spine surgery at the end of follow-up. Cox model with osteopenia status performed significantly better than the model without (p = 0.010). Osteopenic patients had significantly higher risk of surgery (HR2.25, p = 0.011), even after adjustment for menarche status, age and initial Cobb angle. The incremental predictive value of osteopenia was, however, not statistically significant. In the subgroup analysis, cortical bone density was identified as a better marker to improve the sensitivity of the prediction, but requires further larger study to validate this finding. These consistent results of bone density measured at different sites suggest a systemic effect, rather than local effect to the deformed spine, and support to the link of abnormal bone density to the etiopathogenesis in AIS patients.

Adolescent Idiopathic Scoliosis (AIS) is a complex three-dimensional structural deformity of the spine with unknown etiology that commonly occurs in girls aged between 10 and 16^1^,^2^. Prevalence of AIS is about 2–4% worldwide and approximately 10% of those diagnosed with AIS require treatment^1^. The current treatment plan is based on the curve severity measured radiologically as Cobb angle. Immature patients whose Cobb angle < 25° are usually assigned for observation or physiotherapy, following by bracing if curve progresses to 25°–45°. Severe curve with Cobb angle ≥ 45° may require surgery to correct and stabilize the spinal deformity to prevent further deterioration^2^,^3^. The surgical procedure involves fusion and will cause loss of spinal mobility and quality of life^4^.

An accurate prognostic model has the potential to improve the management of scoliosis by identifying patients with high risk of curve progression to benefit from early effective bracing treatment and to avoid over-treatment for those who have low risk. Gender^5^, future growth potential^6^ and magnitude of curve at the time of diagnosis^1^,^2^ are three established predictors of curve progression that are widely used clinically. However, these predictors are only clinical guidelines with limited specificity, particularly in patients presented with mild curves at early stage. Our previous studies showed that about 30% of AIS girls have low bone mineral density (BMD) measured by dual-energy x-ray absorptiometry (DXA)^7^ and BMD was found to be inversely correlated with curve severity during peripubertal period^8^. Longitudinal study revealed that low BMD at the hip was a significant prognostic factor in curve progression with an odds ratio of 2.3^9^. Analysis based on direct bone density measurement of the spine/vertebrae would provide stronger evidence, but due to practical and ethical reasons these measurements were not available in previous studies. We hypothesized the abnormal bone density in AIS patients is systemic, rather that local to the deformed spine. We also hypothesized volumetric BMD (vBMD) is a better assessment than areal BMD (aBMD).

In this cohort study we aimed to evaluate the incremental prognostic value of bone density, assessed at initial clinical visit, on the risk of curve progression beyond maturity. Previous studies commonly defined curve progression as ≥6° increase in Cobb angle^5^,^9^,^10^. A more clinically relevant and important defintion of curve progression: Cobb angle developed to the surgical threshold (≥45°). We tested and validated our hypothesis of the systemic effect of bone density on the risk of curve progression by using one density parameters measured by HR-pQCT at the distal radius in a sub-group analysis.

## Methods

### Study Design and Subjects

This was a longitudinal cohort study of newly diagnosed AIS girls who attended our scoliosis clinic at Prince of Wales Hospital in Hong Kong. All AIS patients, when first presented, were aged between 10 and 16, without prior treatments and had initial Cobb angle ≥ 10°. Exclusion criteria included: (1) patients with initial Cobb angle > 40°, (2) with medical disorders or under medications that were known to affect bone metabolism, or (3) already reached skeletal maturity at the initial assessment.

The study was approved by the Joint Chinese University of Hong Kong–New Territories East Cluster Clinical Research Ethics Committee and was conducted in accordance with relevant guidelines and regulations. Written informed consent was obtained from all patients and their parents before entering the study.

### Data Collection and Definition of Curve Progression

All patients were followed up clinically and radiologically at six months interval until maturity. Maturity was defined as age ≥ 15.5 years and post-menarchal ≥2 years. Standard standing posteroanterior (PA)^11^ radiograph of the whole spine was taken at every clinic visit with Cobb angle measurement and at least one PA whole spine radiograph was taken after reaching maturity to confirm the latest curve status. Age at menarche corrected to the nearest month was recorded. Curve progression to surgical threshold was defined as Cobb angle ≥ 45° and/or had underwent surgery.

### Bone Densitometry Measurements

Because of the presence of axial vertebral rotation in AIS, spinal aBMD measured by DXA is biased and not reliable as reported previously^12^. Hence, aBMD of bilateral proximal femur was measured by DXA (XR-46; Norland Medical Systems, Fort Atkinson, USA) for all subjects within six months of first clinic visit and before any treatments. Standard scanning protocol of proximal femur was used and the details have been described in previous studies^9^,^13^. A normal aBMD dataset of local Chinese girls was used for calculating age- and gender-adjusted z-scores. Osteopenia was defined as z-score ≤−1 at either side of the femoral neck as described in our previous studies^7^,^13^. The short-term precision error of aBMD of femoral neck expressed as coefficient of variation was 1.5%^9^. Bone parameters at the non-dominant distal radius were measured in a subgroup of patients using HR-pQCT (XtremeCT, Scanco Medical AG, Switzerland). Patients were asked to put their forearm in a holding cast to minimize motion artifacts. Repeated scan, at maximum of two times, would be performed if notable motion artifact was seen. The visual grading system suggested by Engelke *et al*. was followed strictly by our operators^11^. Those scans with G4 grade (unacceptable artifacts) were excluded. Reference line was placed at the most proximal limit of the inner aspect of the epiphyseal growth plate and the scan was performed at 5 mm proximal to the reference line^14^. Details of data acquisition have been fully described by Laib *et al*.^15^. HR-pQCT parameters were grouped into three categories: vBMD, bone morphometry and trabecular bone micro-architecture. All scans were done at the first clinic visit and performed by trained densitometry technologists of our Bone Quality and Health Centre.

### Statistical Analysis

The study primary outcome was the time from presentation to the development of curve progression. The time was considered as censored at the last clinic visit for patients whose curve had not reached ≥45° or not received surgery. The Hazard Ratio (HR) of osteopenia status at first clinic visit on curve progression was estimated by fitting Cox’s proportional hazards model. The proportional hazard assumption was tested for all models. Our default model (M0) included covariates age, menarche status (1 = menarche, 0 = pre-menarche) and initial Cobb angle in all analysis. The incremental prognostic value of osteopenia status was studied by comparing the model with osteopenia status to M0. Calibration of models were assessed by Akaike Information Criterion (AIC), Bayesian Information Criterion (BIC) and log-likelihood ratio tests. Area under the curve (AUC) and net reclassification index (NRI) were used to evaluate the discrimination performance between the models. Two AUCs were compared by Delong’s test. To assess the changes in the model sensitivity and specify we used a threshold of 10%, i.e., patients with risk of progression greater than or equal to 10% risk were classified as high risk. This threshold was determined after consulting orthopedic surgeons who considered the potential harm (i.e., surgery) to missed a true positive case. We used bootstrap approach to compute the confidence interval as the 2.5% and 97.5% percentile.

Subgroup analysis was conducted to test and validate the systemic effect of bone density on the prediction of curve progression in AIS patients. We tested the statistical value of incorporating HR-pQCT indicators to DXA aBMD as a composed tool to enhance the predicting ability of the prediction model. With stepwise approach we identified which of the volumetric bone density parameters (trabecular, cortical, and total) provide the best fit of the data. Currently, no designated definition of osteopenia or patients with low bone mass exists for HR-pQCT parameters. As such, Classification and Regression Tree analyses (CART) was used to explore the potential optimal cut-off points of the selected HR-pQCT parameters, aiming to predict curve progression. Age, menarche status and initial Cobb angle were other default variables included in CART. We used the R-package, RPART, to construct the regression tree for the survival data. The tree size was determined by stopping at the split of data that resulted in the smallest error rate estimated by ten-fold cross-validation repeated 100 times.

Since this study aimed to investigate the prognostic value of BMD at initial assessment, subsequent information, such as longitudinal anthropometric measurements, were not included in the prediction model. All these analyses were conducted using R statistical environment.

## Results

### Subject characteristics

A total of 513 eligible subjects were recruited. Of these, 169 (32.9%) subjects were classified as osteopenic and 344 (67.1%) had normal BMD (Table 1). When compared to AIS patients with normal BMD, osteopenic AIS patients had significantly later menarche age (12.2 vs 13.1) and taller in standing height (155.7 cm vs 152.7 cm), at their first clinical presentation. The average follow-up duration was 4.60 (SD 2.05) years. Of all subjects, 54% received brace treatment while 3.5% of them had undergone surgery. Overall, 10.7% of subjects progressed to Cobb angle ≥ 45°. Osteopenic AIS patients (OST) were significantly more likely to progress to ≥45° (17.2% vs 7.6%) or undergone surgery (6.5% vs 2.0%) as compared with AIS patients with normal BMD (Non-OST).

### aBMD added prediction model vs conventional predictive model

Results of univariate and multivariate analyses were similar and consistent with the existing literature: the risk of progression decreased with maturity (age or menarche status) but increased with initial curve magnitude (Table 2). AIS patients with osteopenia were two-fold more likely to progress to surgical level, with (HR = 2.25, 95%CI: 1.20–4.20) or without adjustment of menarche status, age and initial Cobb angle (HR = 2.07, 95%CI: 1.20–3.56). Including osteopenia status to the prediction model improved model fitness significantly as revealed by likelihood ratio test (p = 0.0104), a smaller AIC (528.6 vs 533.13) and BIC (536.6 vs 539.1) (Table 3). Both models reached satisfactory level of AUC (>0.80), whereas model with osteopenia status was slightly better, but not statistically significant (AUC 0.897 vs 0.890). There were no significant changes in terms of sensitivity (0.873 vs 0.873) or specificity (0.793 vs 0.775).

### Subgroup analysis: HR-pQCT

A subgroup of 90 AIS patients was also assessed by HR-pQCT at the initial clinic visit. Seven of them progressed and reached the surgical threshold (Table 4). The cortical area (Ct.Ar), the ratio of cortical area to total area (Ct.Ar/Tot.Ar), cortical vBMD (D_cort_) and trabecular thickness (Tb.Th) of progressed subjects were significantly lower than the non-progressed group.

**Figure 1:**
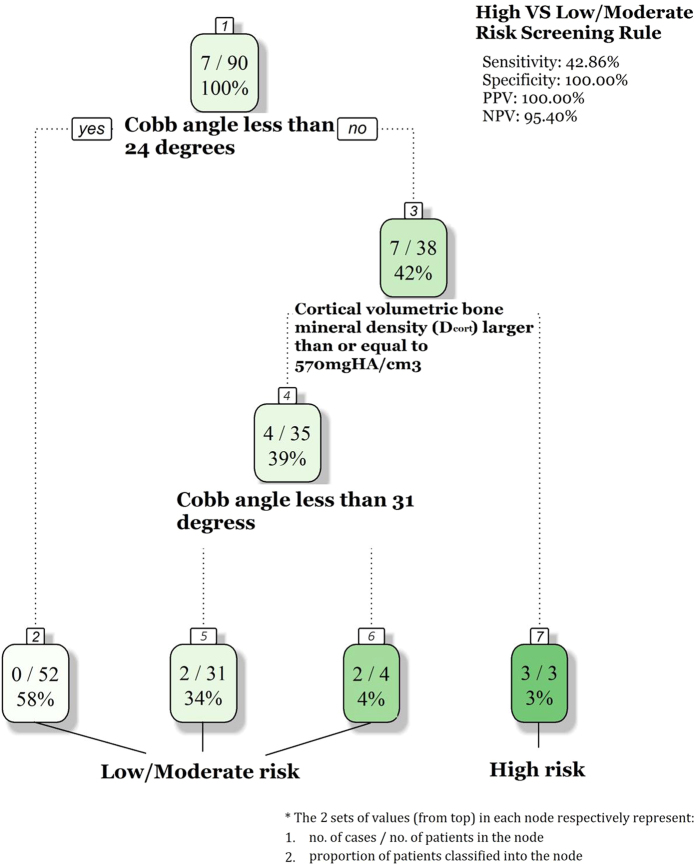
Regression tree generated using available information at first clinic visit including cortical vBMD, initial Cobb angle, baseline age and menarche age as independent variables among 90 patients with HR-pQCT test results. After repeated cross-validations, the model with the smallest error rate was selected where only baseline Cobb angle and cortical vBMD remained as classifiers. Each node in the diagram represents a group of subjects, in which the percentage of subjects progressed to surgery or indication of needs for surgery (Cobb angle ≥ 45°) is shown.

Incorporating the HR-pQCT bone densities to DXA aBMD (M4 vs M1, Appendix Table 1) showed a slight increase in data fitness (both AIC and BIC) and in AUC (from 0.90 to 0.92) with more substantial improvement in sensitivity (0.71 to 0.86) than specificity (0.88 to 0.89). However, these changes failed to reach statistically significant due to limited sample size and events. D_cort_ was identified as the most potential bone density parameter to predict curve progression (M2, Appendix Table 1) and this was further validated by the survival classification tree analysis. For the model building we included the background information (age, Cobb Angle, and menarche status) and all four bone density parameters (one aBMD and three vBMD). The final tree consists of only Cobb angle and D_cort_ (Fig. 1). Subjects were split into three sequential levels based on their initial curve magnitude and Dcot, resulting in four risk groups. The highest risk group consisted of subjects who had initial Cobb angle ≥ 24° and D_cort_ < 570 mgHA/cm^3^. In contrast, subjects with initial Cobb angle < 31° were least likely to progress (2 of 83). Using Cobb angle ≥ 24° and D_cort_ < 570 mgHA/cm^3^ as the cut-off values to define high risk group, provided 43% sensitivity, 100% specificity, 100% PPV and 95% NPV.

## Discussion

Prognosticating curve progression is challenging and important to guide clinical treatment decision in AIS. In this study we investigated the predictive value of BMD, in addition to other well-known risk factors, on curve progression to the surgical threshold (Cobb angle ≥ 45°). Previous prognostic studies mostly defined curve progression as Cobb angle increment ≥6° 9,16. Nevertheless, such increment of Cobb angle may not exert direct clinical significance, e.g., the treatment plan may not be affected even curve progress from 20° to 26°. Using a more critical clinical outcome (reaching surgical threshold), this study demonstrated that osteopenic AIS patients had twice the risk of curve progression than those of having normal BMD, even after adjustment for initial age, menarche status and Cobb angle. Bone density measured in the femoral neck and distal radius was consistently associated with the risk of curve progression – indicating a systemic effect rather than local to the deformed spine only. The incremental predictive value of bone density (both aBMD and vBMD) for curve progression prognosis in the AIS female patients was, however, limited. The main reason is the default model yield a high AUC of 0.89, thus require very large independent associations of the new markers with the outcome, which is rarely observed in epidemiological studies, to result in a meaningful larger AUC^17^. For the volumetric BMD, D_cort_ had the most promising risk classification ability and a cut-off value of D_cort_ < 570 mgHA/cm^3^ at the distal radius with initial Cobb angle ≥ 24° was identified in the sub-group analysis.

Biomechanical loading of both vertebral body and intervertebral disc are hypothesized to affect curve progression in scoliosis patients^18^. Currently, there are no imaging techniques available to measure the stiffness or stress of the intervertebral disc in *in vivo* condition, whereas bone strength is mainly comprised of cortical bone density and the moment of inertia of the bone which are measurable^19^. Due to the rotational deformity of the spine, measurements of lumbar spine BMD are not reliable^12^ and the most accurate technique in assessing BMD at the vertebra or spine is quantitative CT (QCT)^20^. However, high radiation dose and costliness of QCT precludes its clinical use. High to moderate correlation (r = 0.56–0.70) was found in BMD of spine and distal radius measured by different imaging technologies^21^,^22^,^23^. Our ongoing clinical and cadaveric studies correlating bone density parameters measured at the distal radius with QCT measurement of the spine, provide further direct support^24^. Our results indicate that there is a systemic effect of bone density and suggest that AIS patient with lower aBMD at femoral neck and/or D_cort_ at distal radius may imply poor bone mass and strength in vertebra, hence the scoliotic curve could have higher risk of progression. The cortical systemic bone effect might partly be explained by the well-known strong inhibiting power of estrogens upon intra-cortical remodeling^25^, e.g., osteopenia is a generally accepted indicator of estrogen effect on the normal bone growth. Indeed, using the default model (without any bone indicators), menarche status at initial visit was highly associated with curve progression, both in the fulldata analysis (HR = 0.28, p-value = 0.0002) and in the subgroup analysis (HR = 0.22, p-value = 0.067). Thus, the apparent irrelevance of menarche status in the main analysis was explained by the influence of the aBMD z-score value.

While there are strong evidences that BMD of spine and distal radius were correlated, less can be said about other bone parameters, i.e., bone morphometry and bone microarchitecture. Morgan and Keaveny showed different anatomic sites had different trabecular structure^26^. Since vertebral body at spine mainly consists of trabecular bone, it is questionable how well bone morphometry parameters (e.g., trabecular area, cortical area) at distal radius were correlated with bone morphometry at spine. Nevertheless, recent studies showed abnormal bone parameters in AIS manifested by lower cortical and trabecular thickness, lower cortical and trabecular vBMD and more rod-like trabeculae at the distal radius^13^,^14^,^27^. Wang *et al*.^28^ reported the bone geometry, in terms of radius dimension ratio, was associated with curve severity in AIS girls. Stokes *et al*., proposed a “vicious cycle” of scoliosis progression, in which asymmetrical stresses that act on a laterally curved spine and the vertebral growth plates produced asymmetric spinal growth to achieving adequate spine stability^29^. The ‘vicious cycle’ model assumes, however, the mechanically modulated alternation only occur in vertebral bone growth that causes worsening of scoliosis, while everything else (other bone sites) is anatomically and physiologically ‘normal’^30^. In our subgroup analysis of 90 AIS girls and 7 progressed cases, HR-pQCT indicators were however associated with curve progression, especially bone features that were less ‘directional’ (non region-specific) like cortical vBMD. At the first attempt we aimed to test the statistical value of incorporating all HR-pQCT parameters (morphometry, density, and bone microarchitecture) to DXA aBMD as a composed tool to enhance the predicting ability of the analytical model. Due to the relatively small sample size this model did not reach statistical convergence. Results from survival tree analysis showed patients with D_cort_ < 570 mgHA/cm^3^ at the distal radius and initial Cobb angle ≥ 24° were at higher risk of progressing to surgical threshold. Based on these preliminary results, HR-pQCT data could offer some biomechanical explanation for the highly significant role shown for (wrist) cortical bone structure in the determination of (vertebral body) strength. A larger scale study is suggested to optimally explore the potential of HR-pQCT parameters as a useful tool in predicting risk of surgery in AIS patients.

More than 50% of our study cohort required brace treatment according to the usual indication and protocol^4^. Several studies investigated the relationship between BMD and bracing. Sun *et al*.^10^ recently reported that brace treatment would not affect bone mineral accretion and that osteopenia can predict curve progression in braced treated AIS. In our study, we also tried to see if bracing is a confounding factor in predicting risk of surgery. Similar results were obtained after including bracing in the predictive model (HR reduced from 2.24 to 1.97, 95% CI 1.05–3.70). These findings further confirmed poor bone density status plays potentially an important role in curve progression irrespective of whether brace treatment was prescribed or not in growing AIS.

There were several limitations in this study. Only female Chinese AIS patients were included in this study, and hence, the current finding has not yet generalized for all AIS patients. Although AIS is more prevalent in girls, further validation studies in male and other ethnicities should be conducted. Due to ethical concern, we precluded direct QCT measurement of the spine. EOS, the most recent technology that produces high-quality 3D images of the whole spine at low radiation dose, would be a potential alternative imaging system to simultaneously assess BMD and mechanical characteristics of the spine^31^. Despite the longitudinal design, the relationship between osteopenia and risk of progression is still unclear whether it is an association or causation. For instance, progressed AIS patients might be less active and therefore osteopenic. Activity level should be assessed in the future study in order to address this potential confounder. Last, in this study we focused mainly on the density parameters of HR-pQCT to predict curve progression, other secondary parameters, like mechanical properties and the porosity of the cortical bone, could also be investigated in greater detail in future studies.

In conclusion, AIS patients with low bone density had twice or higher risk of curve progression to surgical threshold. This finding was consistent when using aBMD measured by DXA at femoral neck or vBMD of cortical bone measured by HR-pQCT at distal radius, indicating a systemic effect of bone density on the risk of curve progression in AIS. Further multicenter studies with larger scale to explore and validate the clinical usefulness of DXA and HR-pQCT parameters (when available) in different ethnic groups would be essential.

## Additional Information

**How to cite this article**: YIP, B. H. K. *et al*. Prognostic Value of Bone Mineral Density on Curve Progression: A Longitudinal Cohort Study of 513 Girls with Adolescent Idiopathic Scoliosis. *Sci. Rep.* **6**, 39220; doi: 10.1038/srep39220 (2016).

**Publisher's note:** Springer Nature remains neutral with regard to jurisdictional claims in published maps and institutional affiliations.

## Supplementary Material

Supplementary Information

## Figures and Tables

**Table 1:** Baseline characteristics and clinical details of all patients (N = 513).

	Total N = 513	Non-OST N = 344	OST N = 169	P-value #
Demographics, mean (SD)				
Age	13.15 (1.14)	13.16 (1.15)	13.13 (1.12)	0.82
Menarche age	12.49 (1.18)	12.19 (1.09)	13.11 (1.12)	<0.001
Height in centimeters	154.71 (8.96)	155.68 (9.45)	152.73 (7.51)	<0.001
Menarche status (%)	347 (67.6)	270 (78.5)	77 (45.6)	<0.001
Cobb angle in degrees	24.96 (5.69)	24.87 (5.62)	25.13 (5.85)	0.63
Treatment and Clinical Outcomes				
Mean follow-up time in years	4.60 (2.05)	4.44 (2.00)	4.92 (2.11)	0.01
Cobb angle reaching ≥45°, N (%)	55 (10.7)	26 (7.6)	29 (17.2)	0.002
Prescribed treatment, N (%):				<0.001
Observation	236 (46.0)	177 (51.5)	59 (34.9)	
Bracing only	259 (50.5)	160 (46.5)	99 (58.6)	
Bracing and surgery	18 (3.5)	7 (2.0)	11 (6.5)	

OST = Osteopenia (zBMD < −1).

#p-value for t-test of continuous variables and chi-square test of factors.

**Table 2:** Adjusted and unadjusted Hazard Ratio and 95% confidence interval generated from Cox proportional Hazard models among all 513 patients.

	Univariate Analysis				Multivariable Analysis*			
	HR	95% CI		p-value	HR	95% CI		p-value
Menarche status	0.210	0.114	0.386	0.000	0.414	0.197	0.869	0.020
Baseline age#	0.536	0.413	0.696	0.000	0.532	0.377	0.751	0.000
Cobb angle#	1.119	1.068	1.172	0.000	1.155	1.100	1.214	0.000
Osteopenia status	2.068	1.202	3.561	0.009	2.245	1.201	4.198	0.011

^*^Log-likelihood ratio test result showed significant improvement of model with inclusion of osteopenia (Chi-square = 6.5654, DF = 1, p = 0.0104).

^#^Mean-centred.

**Table 3:** Diagnostic capacity of Cox proportional Hazard models with and without osteopenia status based on 5-year prediction of Cobb angle ≥ 45° (indication of needs for surgery).

	AIC	BIC	AUC§ (95% CI)	cNRI (95% CI)
Model without osteopenia status	533.13	539.15	0.890 (0.849–0.931)	0.421 (0.144–0.699)
Model with osteopenia status	528.56	536.59	0.897 (0.859–0.936)	

AIC = Akaike Information Criterion, BIC = Bayesian Information Criterion, AUC = Area Under the Curve, cNRI = Continuous Net Reclassification Index, CI = Confidence Interval.

^§^Area under the curve based on logistic regression.

**Table 4:** Mean (Standard Deviation) of HR-pQCT Parameters (N = 90).

Bone Parameters	Not-Progressed N = 83	Progressed N = 7	p-value (t-test)
*Bone Morphometry*			
Tot Area (cm^2^)	184.1 (32.2)	176.8 (13.7)	0.555
Tb.Ar (cm^2^)	148.9 (33.4)	150.2 (15.7)	0.920
CrAr/TotAr (%)	15 (7.6)	8 (6.3)	0.023
CtPm	54.9 (5.8)	54.61 (2.3)	0.874
CtTh	0.5 (0.2)	0.3 (0.2)	0.015
*Volumetric BMD* (SD)			
D_tot_ (mgHA/cm3)	256.3 (60.3)	212.3 (45.8)	0.063
D_cort_ (mgHA/cm3)	696.6 (77.4)	597.4 (93.2)	0.002
D_trab_ (mgHA/cm3)	145.3 (28.4)	137.9 (26.5)	0.505
*Trabecular Bone Micro-architecture* (SD)			
TrN	1.69 (0.22)	1.82 (0.28)	0.178
BV/TV (%)	12.1 (2.4)	11.5 (2.2)	0.503
Tb.Th	0.07 (0.01)	0.06 (0.01)	0.021
Tb.Sp	0.53 (0.09)	0.50 (0.11)	0.415

BV/TV = bone volume over total volume, CrAr = cortical bone area, CtPm = cortical (periosteal) perimeter, CtTh = cortical thickness, D_cort_ = volumetric density of cortical bone measured at distal radius, D_trab_ = volumetric density of trabecular bone measured at distal radius, D_tot_ = overall volumetric density measured at distal radius, Tb.Ar = trabecular bone area, Tb.Sp = trabecular spacing, Tb.Th = trabecular thickness, Tot Area = total bone area, TrN = trabecular number.
