# Supplementary material for: Prognostic Value of Bone Mineral Density on Curve Progression: A Longitudinal Cohort Study of 513 Girls with Adolescent Idiopathic Scoliosis
Source: Sci Rep. 2016 Dec 19;6:39220. doi: 10.1038/srep39220 (PMC5171643; doi:10.1038/srep39220)
Supplement: Supplementary Information [file srep39220-s1.pdf]

Prognostic Value of Bone Mineral Density on Curve Progression: A Longitudinal Cohort Study of 513 Girls with Adolescent Idiopathic Scoliosis

Benjamin Hon Kei YIP, PhD,[a,c ξ] Fiona Wai Ping YU, MPH,[a,b,d,e ξ] Zhiwei WANG, PhD,[a,b,f ξ] Vivian Wing Yin HUNG, MPhil,[a,b,d,e] Tsz Ping LAM, FRCSED (Ortho),[a,b,d,e] Bobby Kin Wah NG, FRCSED (Ortho),[a] Feng ZHU, MD,[a,b,g] Jack Chun Yiu CHENG, MD,[a,b,d,e *]

[a] *Department of Orthopaedics and Traumatology, Faculty of Medicine, Prince of Wales Hospital, The Chinese University of Hong Kong*

[b] *Joint Scoliosis Research Center of the Chinese University of Hong Kong and Nanjing University*

[c] *School of Public Health and Primary Care, Faculty of Medicine, The Chinese University of Hong Kong*

[d] *Bone Quality and Health Centre, Department of Orthopaedics and Traumatology, Faculty of Medicine, Prince of Wales Hospital, The Chinese University of Hong Kong*

[e] *SH Ho Scoliosis Research Laboratory, Faculty of Medicine, Prince of Wales Hospital, The Chinese University of Hong Kong*

[f] *Department of Orthopedic Surgery, Second Affiliated Hospital, School of Medicine, Zhejiang University, Hangzhou*

[g] *Spine Surgery, the Affiliated Drum Tower Hospital of Nanjing University Medical School, Nanjing, China*

[ξ] ***Co-first authors***

[*] ***Corresponding author***

Address correspondence to Jack Chun Yiu CHENG, MD, FRCSED (Ortho), Department of Orthopaedics and Traumatology, 5/F Lui Che Woo Clinical Sciences Building, Prince of Wales Hospital, Shatin, N.T., Hong Kong.

E-mail: jackcheng@cuhk.edu.hk
Phone: 852-26322729
Fax: 852-26377889

Appendix Table 1 Model comparison with HR-pQCT parameters and aBMD

Models	AIC[a]	BIC[a]	AUC (95% CI)	Sensitivity (95% CI)	Specificity (95% CI)
M0: Default model[c]	49.1	48.9	0.89 (0.79, 1.00)	0.71 (0.43, 1.00)	0.83 (0.75, 0.90)
M1: M0 + aBMD[d]	47.2	47.0	0.90 (0.79, 1.00)	0.71 (0.43, 1.00)	0.88 (0.81, 0.94)
M2: M0 + D_{cort}	46.8	46.6	0.91 (0.78, 1.00)	0.86 (0.57, 1.00)	0.88 (0.81, 0.94)
M3: M0 + D_{cort} + aBMD	47.1	46.8	0.91 (0.77, 1.00)	0.86 (0.57, 1.00)	0.88 (0.81, 0.94)
M4: M0 + D_{cort} + D_{trab} + D_{tot}	48.7	48.4	0.92 (0.79, 1.00)	0.86 (0.57, 1.00)	0.87 (0.79, 0.94)
M5: M4 + aBMD	49.4	49.0	0.92 (0.79, 1.00)	0.86 (0.57, 1.00)	0.89 (0.82, 0.95)

AIC = Akaike Information Criterion, BIC = Bayesian Information Criterion, AUC = Area Under the Curve, CI = Confidence Intervals, aBMD = areal BMD measured at non-dominant femoral neck, D_{cort} = volumetric density of cortical bone measured at distal radius, D_{trab} = volumetric density of trabecular bone measured at distal radius, D_{tot} = overall volumetric density measured at distal radius.

[a]Results based on Cox regression analysis

[b]Used 10% as the cut-off threshold: patients with ≥10% risk were defined as high risk, and below 10% risk as low risk.

[c]Cox regression including covariates: menarche status (Y/N), age and Cobb angle at initial visit.

[d]The aBMD was standardized and the zBMD score was used for the analysis.
